# Different-Shaped Ultrafine MoNbTaW HEA Powders Prepared via Mechanical Alloying

**DOI:** 10.3390/ma11071250

**Published:** 2018-07-20

**Authors:** Yonggang Tong, Peibu Qi, Xiubing Liang, Yongxiong Chen, Yongle Hu, Zhenfeng Hu

**Affiliations:** 1 College of Automobile and Mechanical Engineering, Changsha University of Science and Technology, Changsha 410076, China; yonglehu@126.com; 2 National Engineering Research Center for Mechanical Product Remanufacturing, Academy of Armored Forces Engineering, Beijing 100072, China; qipeibu5200603@163.com (P.Q.); famon1599@163.com (Y.C.); hu_zhenfeng@sina.com (Z.H.); 3 National Institute of Defense Technology Innovation, Academy of Military Sciences PLA China, Beijing 100091, China

**Keywords:** high-entropy alloy, powder technology, microstructure, mechanical alloying

## Abstract

Different-shaped ultrafine MoNbTaW high-entropy alloy powders were firstly prepared by a convenient mechanical alloying method. The phase composition and microstructure of the powders were characterized. The powders are ultrafine with nano-sized grains and a good homogeneous microstructure. All the powders have a single body-centered cubic solid solution phase and form the high-entropy alloy during mechanical alloying. These powders with different shapes are quite attractive for developing high-performance MoNbTaW high-entropy alloy bulk and coatings combined with a following sintering, spraying, or additive manufacturing technique.

## 1. Introduction

Traditional alloying strategy is that ternary or higher-order intermetallic compounds form unexpectedly in multicomponent alloys, which typically result in a reduction in mechanical properties, corrosion resistance, and microstructure stability. Thus, the conventional alloys normally have a base element that dominates the chemistry, even for the superalloys, which contain as many as 12 elements in a single alloy [1,2,3,4]. Recently, a novel alloying concept, multi-principal element alloys or high-entropy alloys (HEAs), has been proposed. The HEAs, composed of five or more elements with near equiatomic ratios, exhibit a simple solid solution microstructure unlike the conventional alloys and have received considerable attention because of their unique properties, such as high hardness and strength, good thermal stability, and excellent corrosion resistance [5,6]. Among them, refractory HEAs are made of refractory elements with high melting temperatures around 2000 °C or higher and implemented for high-temperature applications [7]. Recently, Senkov et al. [8,9] explored a body-centered cubic (BCC)-structured MoNbTaW HEA, attaining a yield strength of 405 MPa and high heat-softening resistance at 1600 °C. Zou et al. [10,11] reported the MoNbTaW HEA thin film and small-sized pillars exhibiting extraordinarily high-yield strengths, which represents a new class of materials in small-dimension devices potentially for high-stress and high-temperature applications. Thus, the attractive performance of the MoNbTaW HEA has drawn more and more attention. Until now, the refractory HEAs have mostly been prepared by the arc melting technique, which has been widely studied in the published literature [12,13,14]. Because the alloys contain multiple elements, it is hard to avoid the compositional segregation of high-melting elements and the evaporation of low-melting elements. In addition, HEA ingots have limited shapes and sizes with dendritic microstructures. There is a high demand to explore new fabrication methods for refractory HEAs. Mechanical alloying is a practical method to synthesize the nanocrystalline and ultrafine powders. Bulk ultrafine-grained materials with large sizes and various shapes can be fabricated, combined with a following sintering, spraying, or additive manufacturing technique. There have been several attempts to synthesize HEAs using the mechanical alloying method. Mohanty et al. [15] synthesized AlCoCrFeNi high-entropy alloy by high-energy ball milling and characterized the microstructure of the as-produced powder. Cai et al. [16] prepared a NiCrCoTiV HEA powder by mechanical alloying and then fabricated the bulk HEA alloy by vacuum hot-pressing sintering. Joo et al. [17] produced an ultrafine-grained CoCrFeMnNi HEA by mechanical alloying and spark plasma sintering. The previous investigations on the mechanical alloying of HEAs are mainly focused on the late transition metals, such as Fe, Co, Cr, Al, Cu, and Ni. Very little research is available on the refractory HEAs. Regarding the excellent performance of the MoNbTaW HEA, mechanical alloying was proposed to prepare the MoNbTaW HEA powder in the present investigation. Near-spherical and lamellar-shaped MoNbTaW HEA powders were prepared and the microstructure of the powders were characterized.

## 2. Materials and Methods

Pure elemental powders, Mo, Nb, Ta, and W, commercially available from Shanghai Naiou Nanotechnology Co. Ltd. (Shanghai, China), with high purity (>99.9 wt %) and uniform particle size (300 mesh) were used as the starting materials. The starting elemental powders were mechanically alloyed using a planetary ball milling machine (TENCAN, Changsha, China) in a protective argon (Ar) atmosphere with steel vials and balls. In order to obtain a homogeneous microstructure and composition, the vials were horizontally circumgyrated at the same time of turning at 360 degrees. The powders were milled for up to 60 h at the speed of 300 rpm with a ball-to-powder ratio of 10:1. Prior to the milling, the vials containing the powder and balls were evacuated and refilled with high purity (99.9%) argon gas. The milling operation was stopped 10 min after running every 20 min to ensure the protective atmosphere was retained inside the vials during milling. Different process control agents, including ethanol and stearic acid, were added during the mechanical alloying process. The as-milled powders were annealed at 1200 °C for 2 h in the Ar atmosphere to characterize the stability of the as-milled powders. The micro-morphologies of the HEA powders were observed by scanning electron microscopy (SEM, FE-SEM, Zeiss Ultra55, Zeiss, Oberkochen, Germany) operating at 15 keV. Phase composition and crystal structures were examined using an X-ray diffractometer (XRD, RIGAKU D/MAX-2500, Rigaku, The Woodlands, TX, USA) with Cu–Ka radiation. The chemical composition was observed by energy dispersive X-ray (EDS) equipped in the SEM. The structure of the powders was analyzed in transmission electron microscopy (TEM, FEI, Hillsboro, OR, USA). The samples were prepared by placing a drop of the milled powder solution on a carbon-coated copper grid and then drying it at room temperature.

## 3. Results and Discussion

**Figure 1:**
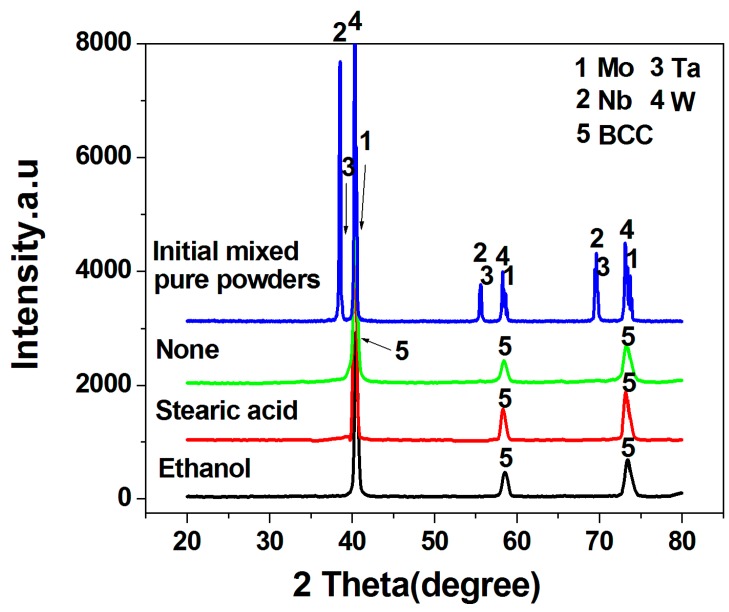
XRD patterns of the initial mixed pure powders and the MoNbTaW high-entropy alloys (HEA) powders prepared by mechanical alloying with different process control agents.

Figure 1 shows the XRD patterns of the initial mixed pure powders and the MoNbTaW HEA powders prepared by mechanical alloying with different process control agents. The initial mixed pure powders exhibit the XRD patterns of Mo, Nb, Ta, and W. The patterns of Nb are quite close to that of Ta, and the patterns of W are quite close to that of Mo. All the as-milled powders are composed of a unique BCC solid solution, indicating that high-entropy alloys have been formed without any residual pure elements. Crystallite size, lattice constant, and strain of the as-milled powders were calculated based on the XRD data and listed in Table 1. As shown, the crystallite size of the powder prepared without any process control agents is the smallest, 11.8 nm, which is much smaller than that prepared with ethanol or stearic acid, 24.2 and 14.7 nm, respectively. In addition, the strain of the powder prepared without any process control agents is the largest. The above results indicate that dry balling without process control agents has the highest milling energy. During mechanical alloying, flattening, cold-welding, fracturing, and rewelding occurred repeatedly, and the force of the impact severely deformed the powder particles [18]. The elements in the starting powders diffused fast, and the size of the powders was gradually refined [18,19]. Finally, a relatively thermodynamically stable powder with fine grains was formed.

The BCC solid solution structure formation of the MoNbTaW alloy during mechanical alloying could be explained by the high-entropy effect in the system. The mixing entropy (ΔS) of the equiatomic MoNbTaW alloy calculated by Equation (1) [20] is 11.5 J/(K·mol), which is muchlarger than that of a traditional binary alloy (5.64 J/(K·mol)). According to the Gibbs free energy formula, G_mix_ = H_mix_ − TS_mix_, free energy of the system will be smaller with higher mixing entropy, and thus, the solid solution is more favorably formed [21]. Furthermore, several parameters, such as mixture enthalpy (ΔH) [20], atomic size mismatch (δ) [22], valence electron concentration (VEC) [23], and thermodynamic parameter (Ω) [20] are employed to predict the phase formation and stability of single-phase solid solution in HEAs. They are defined and calculated based on the following equations:

(1) $$\Delta S=-R\overset{n}{\underset{i=1}{\sum }}c_{i}{\mathrm{lnc}}_{i}$$

(2) $$\Delta H=4\overset{n}{\underset{i=1,\;i\neq j}{\sum }}\Delta H_{\mathrm{ij}}^{\mathrm{mix}}c_{i}c_{j}$$

(3) $$\delta =100\sqrt{\overset{n}{\underset{i=1}{\sum }}c_{i}\left(1-r_{i}/\overset{n}{\underset{i=1}{\sum }}c_{i}r_{i}\right)}$$

(4) $$\mathrm{VEC}=\overset{n}{\underset{i=1}{\sum }}c_{i}{\left(\mathrm{VEC}\right)}_{i}$$

(5) $$\Omega =\frac{\Delta S_{\mathrm{mix}}}{\left|\Delta H_{\mathrm{mix}}\right|}\overset{n}{\underset{i=1}{\sum }}c_{i}{\left(T_{m}\right)}_{i}$$

where R is the gas constant, $\Delta H_{\mathrm{ij}}^{\mathrm{mix}}$ is the mixture enthalpy of the *i*th and *j*th components, c_i_ and c_j_ are the atomic percentage of the *i*th and *j*th components, respectively, r_i_ is the atomic radius of the *i*th component, (VEC)_i_ is the VEC of the *i*th component, and (T_m_)_i_ is the melting temperature of the *i*th element. Normally, the formation of simple BCC solid solutions in HEAs is concluded: −22 ≤ ΔH_mix_ ≤ 7 kJ·mol^−1^, 0 ≤ δ ≤ 6.6, VEC < 6.87, and Ω > 1.1 [20,22,23]. The above parameters ΔH, δ, VEC, and Ω for the MoNbTaW alloy in the present investigation were calculated to be −6.5 KJ/mol, 2.78, 5.5, and 5591, respectively, well meeting the criterion for the formation of a single BCC solid solution structure. Therefore, the MoNbTaW alloy tends to form a BCC single-phase structure rather than a complex structure alloy during mechanical alloying.

**Figure 2:**
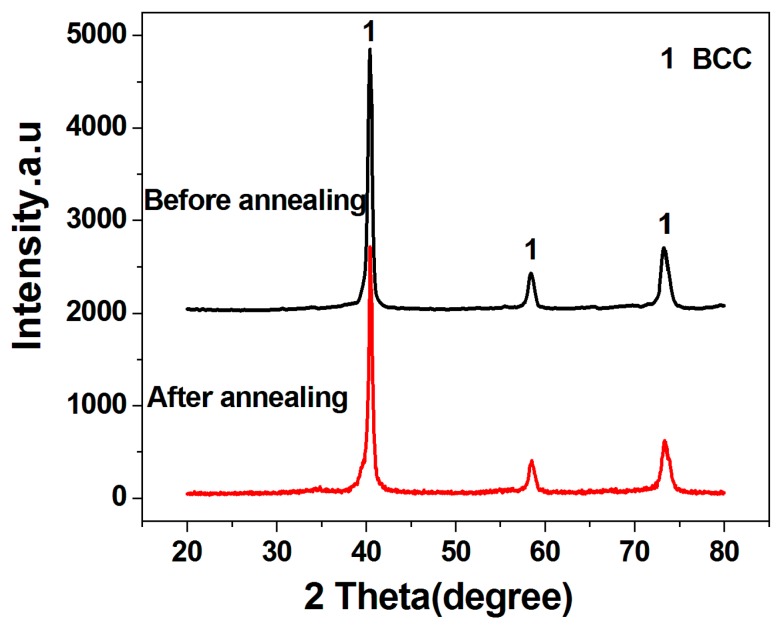
XRD patterns of the HEA powders prepared without process control agents before and after annealing at 1200 °C.

Figure 2 shows the XRD patterns of the as-milled HEA powders prepared without process control agents and the powders annealed at 1200 °C. No obvious change was found before and after annealing, demonstrating that the HEA powders have a good stability. As mentioned above, the mixing entropy (ΔS) of the MoNbTaW HEA alloy is much larger than that of the traditional binary alloys, which may be the main reason for the good phase stability of the HEA powders [21].

**Figure 3:**
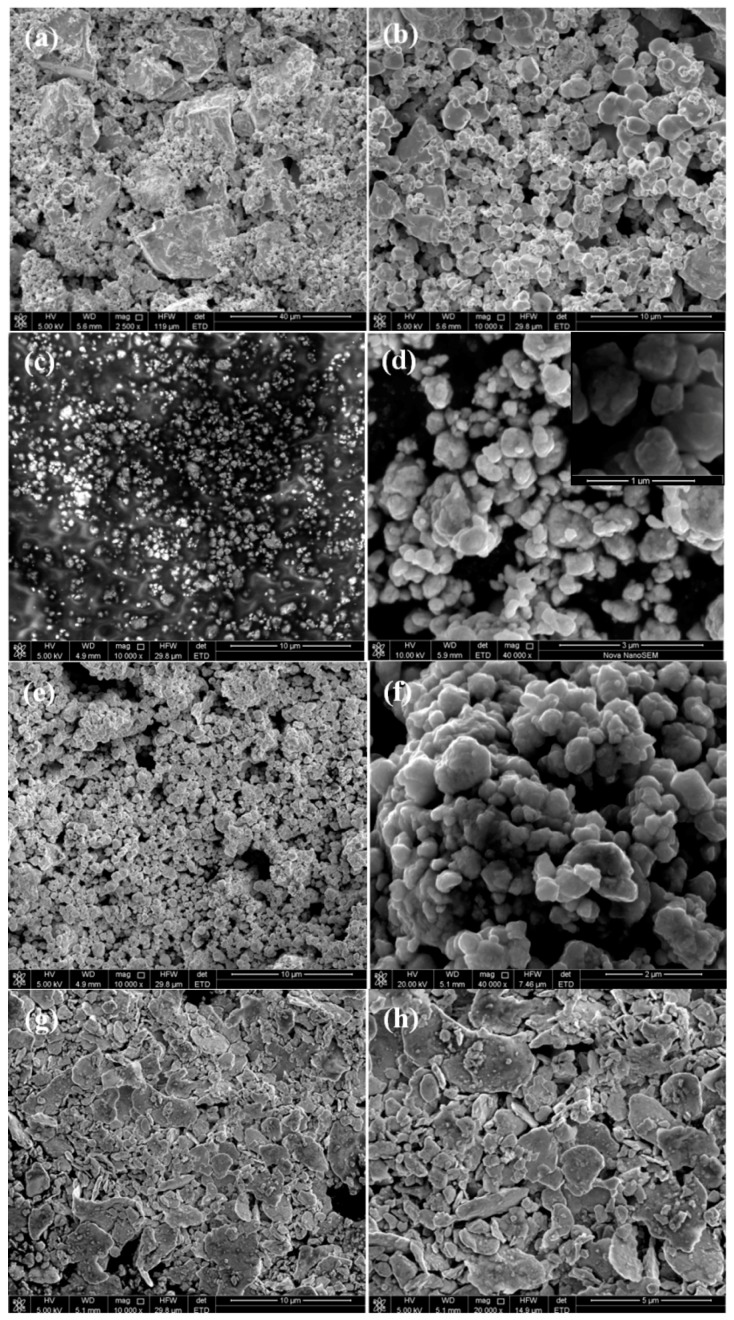
Micro-morphologies of the starting powder and the MoNbTaW HEA powders prepared by mechanical alloying with different process control agents, (**a**,**b**) the starting powder, (**c**,**d**) without process control agent, (**e**,**f**) stearic acid, and (**g**,**h**) ethanol.

Figure 3 shows the micro-morphologies of the starting powder and the MoNbTaW HEA powders prepared by mechanical alloying with different process control agents. As can be seen, the starting powder is composed of nonuniform powders with different shapes and sizes. After mechanical alloying, all the powders show relatively homogeneous sizes and shapes. The HEA powders prepared with stearic acid and without any process control agents have a near-spherical shape, while the powder prepared with alcohol exhibits a lamellar morphology. Detailed morphologies of the powders show that the spherical powders are composed of many nano-crystalline grains (Figure 3d), which is consistent with the XRD analysis.

**Figure 4:**
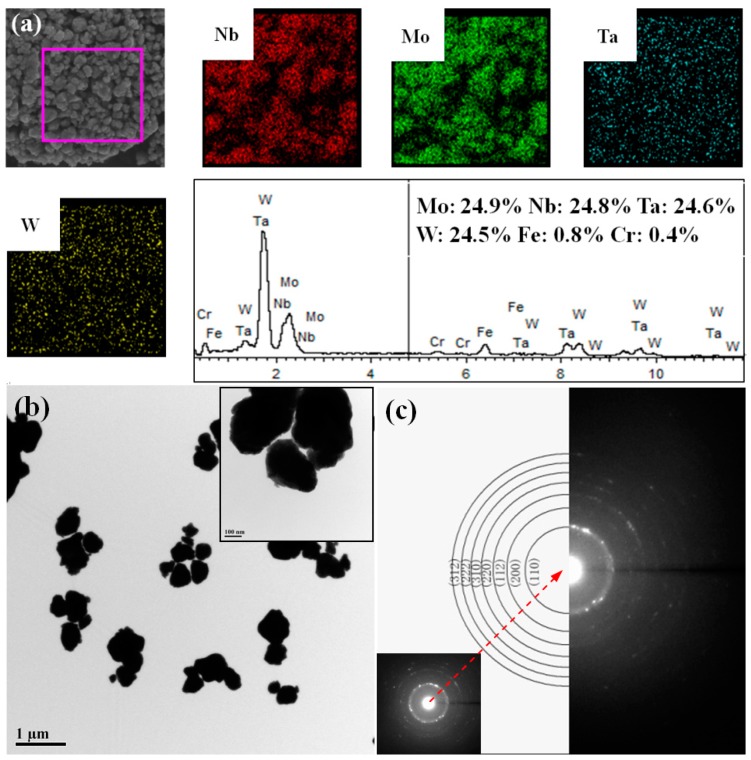
(**a**) EDS analysis of the MoNbTaW HEA powders milled without process control agent, (**b**,**c**) TEM image of the MoNbTaW HEA powders with the selective electron diffraction patterns.

Figure 4 shows the EDS analysis of the MoNbTaW HEA powders. As can be seen, the powder is composed of approximately equiatomic Mo, Nb, Ta, and W elements in addition to very little amounts of Fe and Cr (total 1.2 at %). These Fe and Cr elements should be from the steel vials and balls during the mechanical alloy process. However, their amounts are very little, indicating that the HEA powders were not obviously contaminated by the steel vials and balls under the present mechanical alloying process. The tiny content of Fe and Cr may be the reason why the Fe and Cr diffraction peaks were not detected in the XRD pattern in Figure 1. The mapping EDS analysis of the powder shows a uniform distribution of the Mo, Nb, Tam and W elements, further demonstrating the homogenous formation of the HEAs.

TEM analysis was performed to further characterize the microstructure of the MoNbTaW HEA powders, and the results are shown in Figure 4. Selected area electron diffraction (SAED) analysis of a small powder exhibits a polycrystalline ring diffraction of the BCC structure, indicating that the powder is composed of very small nano-crystalline grains. These results are in good agreement with the XRD analysis in Table 1 and further confirm the BCC solid solution structure of the HEA powders prepared by mechanical alloying.

## 4. Conclusions

Mechanical alloying is demonstrated to be a practical fabrication process for uniform MoNbTaW HEA powders. Near-spherical and lamellar-shaped HEA powders with a good homogeneous microstructure and ultra-fined grains were prepared via mechanical alloying, respectively. The powders are ultrafine and have a single BCC solution structure.

## Figures and Tables

**Table 1:** Crystallite size, lattice constant, and strain of the MoNbTaW powders prepared with different process control agents.

Powders Prepared with Different Process Control Agents	Crystallite Size (nm)	Lattice Constant (Å)	Strain (%)
None	11.8	3.1636	0.688
Ethanol	24.2	3.1549	0.483
Stearic acid	14.7	3.1648	0.590
